# Supplementary material for: Patterns of genetic differentiation and the footprints of historical migrations in the Iberian Peninsula
Source: Nat Commun. 2019 Feb 1;10:551. doi: 10.1038/s41467-018-08272-w (PMC6358624; doi:10.1038/s41467-018-08272-w)
Supplement: Supplementary file 1 — Supplementary Information [file 41467_2018_8272_MOESM1_ESM.pdf]

Supplementary Information

Patterns of genetic differentiation and the footprints of historical migrations in the Iberian Peninsula

Clare Bycroft[1], Ceres Fernandez-Rozadilla[2], Clara Ruiz-Ponte[2], Inés Quintela[3], Ángel Carracedo[2,3,4], Peter Donnelly[1,5†], Simon Myers[5,1,†‡]

1) Wellcome Trust Centre for Human Genetics, University of Oxford
2) Fundación Pública Galega de Medicina Xenómica- CIBERER-IDIS, Santiago de Compostela
3) Grupo de Medicina Xenómica, Centro Nacional de Genotipado (CEGEN-PRB2-ISCIII). Universidade de Santiago de Compostela
4) Institute of Forensic Sciences. University of Santiago de Compostela
5) Department of Statistics, University of Oxford

† These authors jointly directed this work.
‡ To whom correspondence should be addressed: myers@stats.ox.ac.uk

Supplementary Notes **3**

Note 1 **Details of quality control and phasing** **3**
1.1 Spanish cohort data 3
1.2 Non-Spanish data 3

Note 2 **Details of fineSTRUCTURE analyses** **5**
2.1 Rationale for using total length of genome as coancestry measure 7
2.2 Estimating the fineSTRUCTURE *c*-factor 9
2.3 Discussion of potential sampling effects 10

Note 3 **Map-based data visualisation** **11**

Note 4 **Investigation into ultra-fine-scale structure in Galicia** **12**

Note 5 **Signals of drift and admixture in coancestry matrices** **15**

Note 6 **Details of defining non-Spanish donor groups** **16**

Note 7 **Details of estimating ancestry profiles** **17**
7.1 Cluster-based ancestry profiles 17
7.2 Spatially smoothed ancestry profiles 20
7.3 Discussion of ancestry profile results 22

Note 8 **Details of GLOBETROTTER analyses** **25**
8.1 Details of the two analyses 25
8.2 Evaluating the statistical support for inferred admixture events and different scenarios 28

Note 9 **Testing association of historical linguistic regions with genetic clusters** . **31**

Supplementary References **37**

Supplementary Notes

Note 1 Details of quality control and phasing

1.1 Spanish cohort data

In the Spanish cohort data, 1,548 individuals (before QC) were genotyped on the Affymetrix 6.0 chip (~900,000 SNPs) and we used the output of the genotype calling algorithm (Birdseed [1]) as the starting point in our analysis. The genotype calls were coded using a mixture of forward and reverse strands, so we first flipped all SNPs to the positive strand and converted the SNP positions to hg19 coordinates. This could be done with certainty by using the appropriate Affymetrix 6.0 chip annotation file (Release 34), and using the software Birdsuite (v1.4) [1].

We set to missing all Birdseed genotype calls with a 'confidence' value greater than 0.1, and then excluded SNPs that had one or more of the following properties:

- Departure from Hardy-Weinberg equilibrium with p-value < 10^{-20}.
- Minor allele frequency < 0.01.
- Missing rate > 0.02.
- On the Mitochondrial, Y, or X chromosomes.
- Its rsID did not map to genome assembly build hg19.
- Did not match the strand of the reference panel used in phasing.

We also excluded samples with one or more of the following properties, where all metrics were calculated after excluding SNPs as described above.

- Genome-wide missing rate > 0.009 (79 samples)
- Born outside of Spain, or at least one grandparent born outside Spain (where information available) (21 samples)
- Outliers in the first two principal components (7 samples).
- Kinship co-efficient > 0.1 (excluded one from each related pair), computed using KING v1.4 [2] (9 samples).
- Showed signs of poor quality genotyping after running fineSTRUCTURE (19 samples) (discussed later).

After applying the above quality control filters (using *qctool* v1) there remained 1,413 samples and 693,092 SNPs for further analysis. We phased the quality-filtered genotype data using SHAPEIT v2 [3] with a reference panel and recombination map from Phase I 1000 Genomes [4]. Related samples were included in the phasing step, but excluded from the fineSTRUCTURE analysis.

1.2 Non-Spanish data

For the analysis involving individuals from outside of Spain, we first switched all genotypes to the positive strand; shifted to genome build hg19 coordinates and excluded SNPs not in the intersection of the two arrays. We merged all four data

sources (including the un-phased genotypes for the Spanish cohort described above) using the *plink* (v1.7) 'merge' function [5]. This formed a dataset of 359,247 SNPs and 6,954 samples prior to QC and phasing.

Combining data from multiple sources presents unique quality control issues, such as strand misalignments, or subtle biases due to the genotyping taking place in different laboratories and on different arrays. For instance, we looked for large differences in MAF between each data source and the Spanish cohort, which might indicate strand misalignments; and we excluded SNPs based on MAF computed for each data source separately. These considerations must be balanced with the need to retain enough SNPs for haplotype-based analyses. Specifically, we excluded SNPs with one or more of the following properties:

- MAF < 0.01 within at least one data source.
- Departure from Hardy-Weinberg equilibrium with p-value < 10^{-20} (computed in Spanish cohort only).
- Overall sample missing rate > 0.05
- MAF in at least one data source differs from MAF in the Spanish cohort by more than 0.2, 0.4 and 0.6 for POPRES, north Africa, and Hapmap3 respectively (values are based on visual examination of the distribution of MAF differences for the different groups).
- Mismatch with the strand of the reference panel used in phasing.
- SNP did not map to genome assembly build hg19.

We also excluded 221 samples with a call rate (on the filtered SNPs) less than 95% in at least one chromosome, as this is a requirement for phasing. The merged dataset of 6,617 individuals was phased altogether using SHAPEIT v2, and with the same reference panel and recombination map as the Spanish-only dataset. After phasing we excluded samples using the following criteria:

- A child in a reported trio, or one sample of a related pair with kinship > 0.1, computed using KING v1.4 [2] (excluded the sample with the lowest call rate)
- POPRES individuals labelled as non-European or of mixed ancestry
- POPRES individuals with non-European ancestry based on a principal components analysis.
- Spanish individuals in POPRES (the field `GROUPING_PCA_LABEL1' = 'Spain').

For POPRES individuals we also required that all four grandparents were born in the same country, except for individuals from Ireland and the UK as there was no grandparental birth-place information available. In POPRES there are many more individuals from UK and Switzerland than from other parts of Europe (~1,000) from Switzerland; ~400 from the UK). To ensure more even sampling across Europe we picked 90 samples from each of the sets of samples labelled 'Swiss-French' and 'Switzerland' based on the field 'GROUPING_PCA_LABEL1' provided in the POPRES auxiliary data. This left 2,969 individuals and 300,895 SNPs in the merged dataset, which we used in our main analyses.

Note 2 Details of fineSTRUCTURE analyses

We ran several fineSTRUCTURE (v0.0.5) analyses using phased haplotypes from the following sets of individuals:

(A) Spanish individuals
(B) Spanish and Portuguese individuals
(C) Non-Spanish individuals

In all cases we used CHROMOPAINTER software (v2) [6] to estimate the coancestry matrix. Briefly, for each haplotype *i* CHROMOPAINTER applies a hidden Markov model (HMM) where the hidden state at each locus (SNP) is the haplotype *j* with which haplotype *i* shares a common ancestor which is the most recent at that locus (haplotype *j* cannot belong to the same diploid individual as haplotype *i*). We ran CHROMOPAINTER using the same recombination map as we used in phasing. Two parameters are required for the HMM: the initial genome-wide average 'switch' rate (n) and global 'emission' rate (M). For analysis (A) we estimated these using 10 iterations of CHROMOPAINTER's Expectation-Maximization (E-M) algorithm, across 10 individuals chosen from a variety of regions across Spain, but allowing all other Spanish individuals to be donors. We then averaged the results over all chromosomes and the 10 individuals using the auxiliary program ChromoCombine. For each of analyses (B) and (C) we re-estimated these parameters in a similar manner, using a subset of samples from a variety of regions in each case. This procedure mimics that used by a previous successful application of fineSTRUCTURE [42]. All other parameters were set to the software defaults.

In all analyses, the *c*-factor parameter required for fineSTRUCTURE's MCMC algorithm was computed as described in Note 2.2. Also for all cases we used 500,000 burn-in iterations and 1,000,000 subsequent iterations, and stored the results from every 10,000th iteration. The MCMC was followed by 100,000 hill-climbing moves before applying fineSTRUCTURE's tree-building algorithm. In analysis (A) we also conducted an iterative procedure after the MCMC and hill-climbing iterations to further refine the final set of clusters at the bottom of the tree. This is described in full in [7]. Informally, it uses an iterative procedure to reassign individuals to a new set of clusters, such that individuals that are often co-clustered across multiple MCMC samples share a cluster assignment. We used the last 50 stored iterations of the MCMC, as these showed no signs of increasing posterior probability. We also checked that the MCMC samples were independent of the algorithm's initial position by visually comparing the results of two independent runs starting from different random seeds. Good correspondence in the pairwise coincidence matrices of the two runs indicates convergence of the MCMC samples to the posterior distribution [6] (**Supplementary Figure 1a**). We used the first of these two runs in the main analysis.

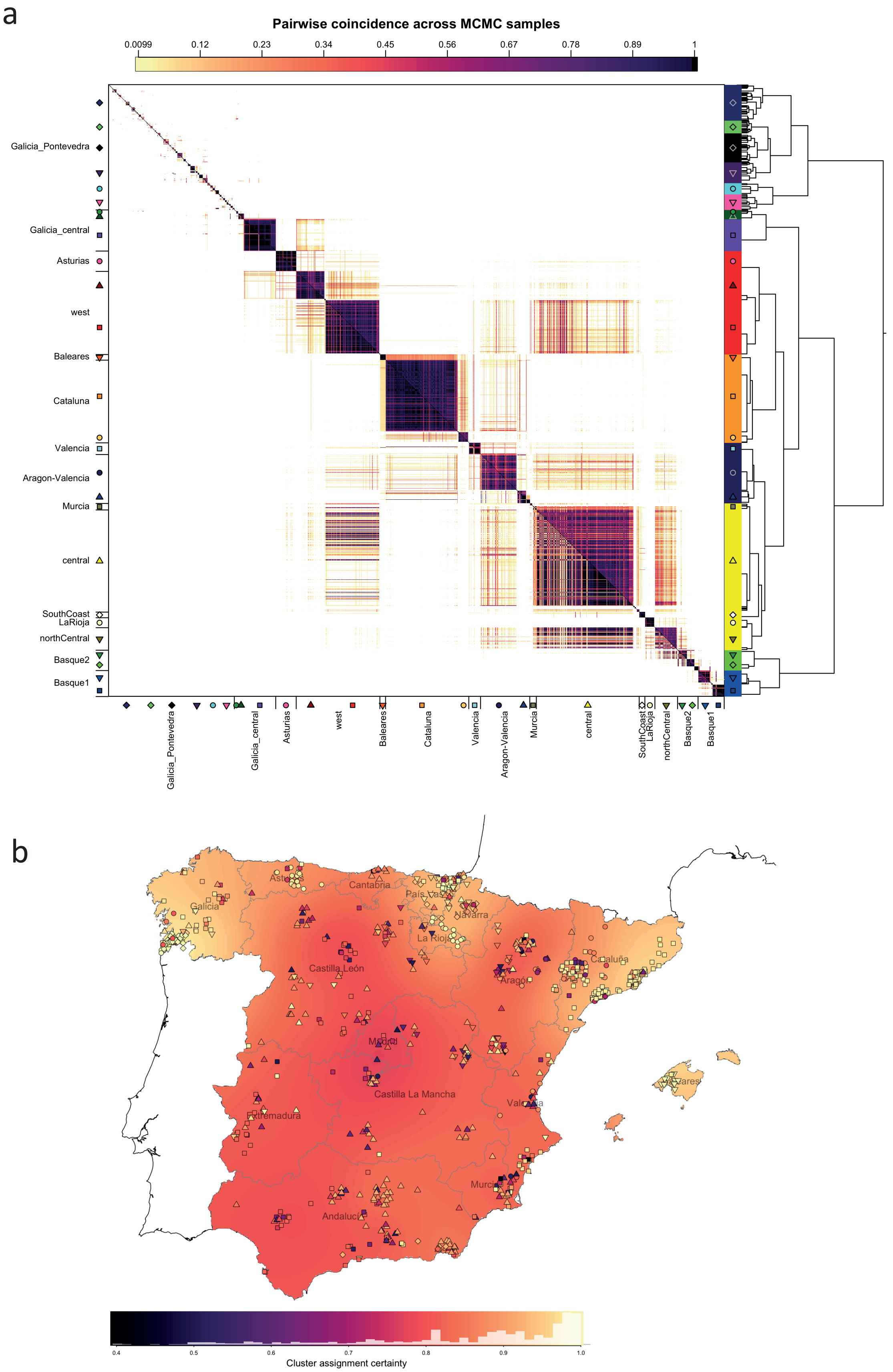

Supplementary Figure 1 | Convergence of fineSTRUCTURE MCMC and cluster assignment certainty (a) Pairwise coincidence of cluster assignments for two independent fineSTRUCTURE runs. We measured the fraction of times that a pair of individuals (row and column) are assigned to the same cluster across the set of MCMC samples used to construct the final set of clusters (Methods). White (0) indicates no co-assignment across all MCMC samples, and black (1) indicates perfect co-assignment across all MCMC samples. The upper triangle shows results for analysis (A) and the lower triangle shows results for an independent run using exactly the same input data and parameters but with different a random seed. The sample ordering and tree are from analysis (A). The clear similarity between the two runs indicates convergence of the MCMC samples to the posterior distribution. **(b)** Cluster assignment certainty for analysis (A). For the finer level of the tree shown as points in **Figure 1b** (27 clusters) we computed a measure of cluster assignment certainty which measures the co-clustering of individuals over multiple MCMC samples (1 is high certainty) (Methods). Points have been coloured according to this certainty measure computed for each individual, and the symbols match those shown in **Figure 1b** to distinguish between different clusters. The histogram shows the distribution of the certainty measure for the individuals shown on the map. The background colour has been determined by applying a spatial smoothing algorithm to the same data (Methods).

2.1 Rationale for using total length of genome as coancestry measure

The CHROMOPAINTER algorithm computes two summaries of ancestry sharing between individuals in a sample: the number of chunks of the genome for which an individual *i* shares its most recent common ancestor with individual *j*; and the total amount of genome (measured in cM), for which an individual *i* shares its most recent common ancestor with individual *j*. An observation made in [6] is that individuals with relatively high levels of recent common ancestry will tend to share more chunks (relative to other individuals in the sample), but these chunks will also tend to be longer, because there has been less time for recombination to break up the shared chunks. Therefore, if clusters inferred under the fineSTRUCTURE model are capturing groups of individuals that are genuinely more closely-related, we would expect a positive correlation between the number of chunks, and the average length of the chunks shared between individuals within clusters. We exploited this property to identify spurious cluster assignments that were likely a result of poor data quality. Intuitively, more genotyping errors would increase the number of 'switches' occurring in the HMM used to estimate the coancestry matrix, thus inflating the number of chunks copied. The total amount of genome coancestry measure should be less sensitive to this property because it is a sum over all chunks copied from the same donor (regardless of spurious switches in the HMM), so would still capture signals of real ancestry sharing despite the noise due to genotyping errors.

Supplementary Figure 2a and **b** shows results from a fineSTRUCTURE analysis using chunk counts as the coancestry measure. Specifically, there is a set of clusters with individuals who share high numbers of chunks relative to other clusters, but share unexpectedly short chunks on average. These same clusters also have significantly higher sample missing rates than other clusters (**Supplementary Figure 2b**), a symptom of poor genotype quality. We found that this effect was reduced when using the total amount of genome as the coancestry measure (**Supplementary Figure 2c** and **d**). In that case, the extent of chunk count inflation was much reduced, with only 19 individuals that showed evidence of spurious cluster assignment, compared to 89 when using chunk counts (with exactly the same algorithm parameters, except a different *c*-factor).

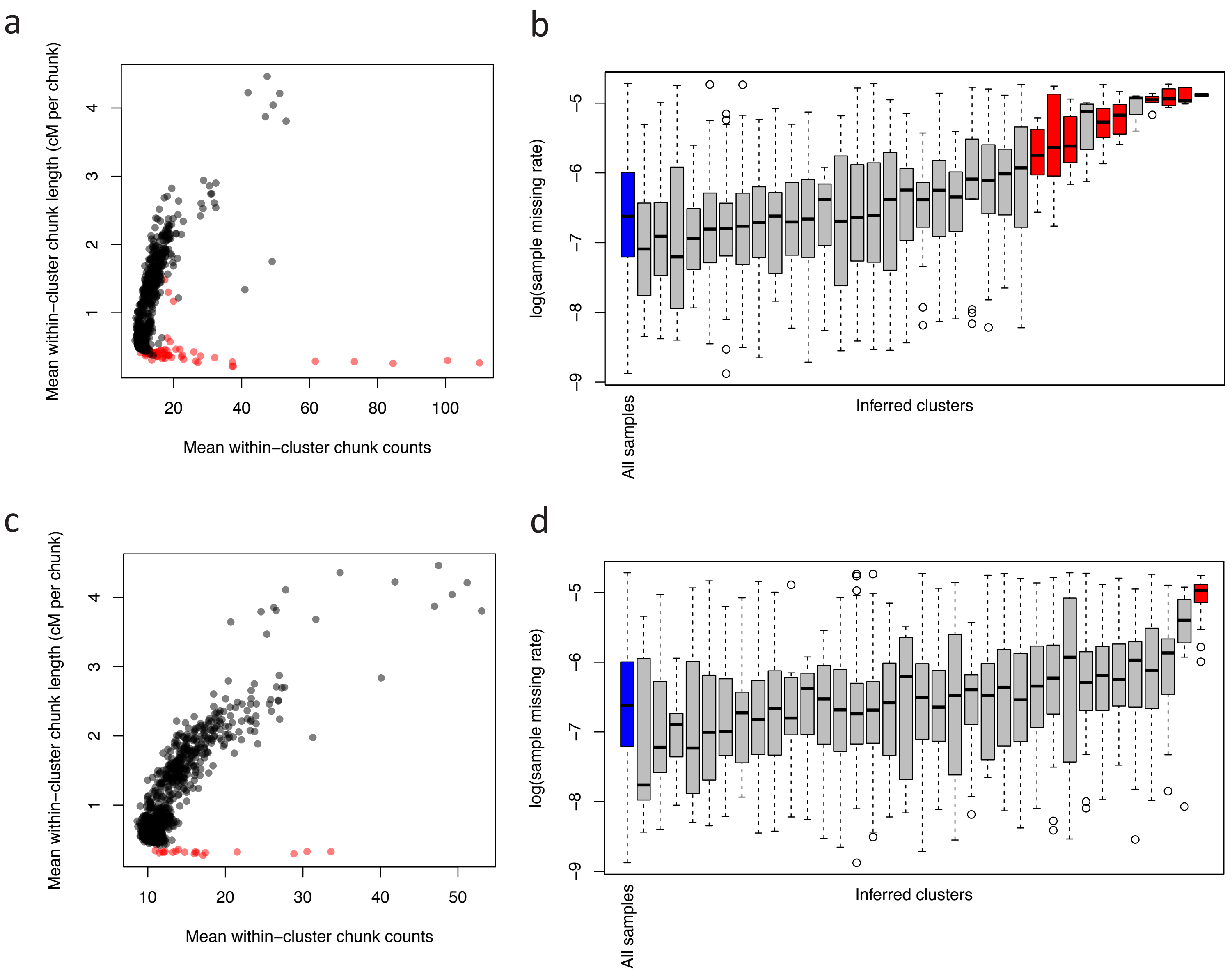

Supplementary Figure 2 | Effect of using total lengths verses chunk counts as coancestry measure in fineSTRUCTURE algorithm. We ran fineSTRUCTURE for the Spanish cohort using two different coancestry measures, and compared their robustness to genotype quality (Methods). Plots **(a)** and **(b)** show results from a fineSTRUCTURE analysis using chunk counts as the coancestry measure. Plots **(c)** and **(d)** show results from a fineSTRUCTURE analysis using total amount of genome copied as the coancestry measure. For both runs we show these metrics for the level of the hierarchical tree with 35 clusters. The left-hand plots (a) and (c) show, for each individual, the mean chunk lengths (i.e. the average length of copied chunks), and mean number of chunks copied from other individuals inferred to be part of the same cluster. The right-hand plots (b) and (d) show the distribution of genotype missing rates (on the log scale) for the samples in each of the inferred clusters. In all plots, the clusters with significantly higher missing rates from the overall cohort are shown in red ($p < 0.001$, one-sided t-test on log-transformed sample missing rates).

2.2 Estimating the fineSTRUCTURE *c*-factor

Recall that fineSTRUCTURE applies a MCMC procedure to find a high probability partition of individuals into any number of clusters. The posterior probability of a given partition is inferred using the following multinomial likelihood function (other details such as priors are in [6]):

$$F(x|p,q) = \prod_{i=1,j=1}^{N} \left(\frac{P_{q_i q_j}}{\hat{n}_{q_i}}\right)^{\frac{x_{ij}}{c}}, \qquad (1)$$

where x_{ij} is the measured coancestry between individual *i* and *j*; q_i and q_j denote the clusters that individual *i* and *j* are assigned to; $\hat{n}_{q_i}$ is the number of samples assigned to cluster q_j; and $P_{a,b}$ is a cluster-level coancestry matrix. That is, the proportion of coancestry donated to any individual in cluster *a* from any individual in cluster *b*. This multinomial model implies that each unit of coancestry (either number of chunks, or cM) that contributes to the coancestry matrix is an independent draw from a set of possible outcomes, where an outcome is a particular donor haplotype. In practice, the independence assumption does not always hold, and so the coancestry matrix should be scaled by a factor of c to account for this. The authors of fineSTRUCTURE recommend estimating this value (and show that it is a good approximation to that predicted by theory [6]) by computing the ratio of the empirical variance of the coancestry values, to the theoretical variance assuming the data is generated from the multinomial distribution. The fineSTRUCTURE software automatically calculates *c*, but because we used a different measure of coancestry we also estimated it differently.

Specifically, we measured the empirical variance of the coancestry values in a similar fashion to that used by fineSTRUCTURE software by breaking up the genome into segments of equal length, and which are long enough to be approximately independent. Since we used total genome length as the coancestry measure we used segments of a length defined in cM rather than number of chunks. We used 40 cM as there would be sufficient recombination (by definition) between SNPs this distance apart such that linkage disequilibrium across segments would be minimal; and given that the average length of chunks in the Spanish cohort tended to be about 0.5 cM, a segment size of 40 cM corresponds approximately to the size (100 chunks per segment) noted by the authors as working well in real human data sets. This distance is also small enough such that all chromosomes have at least one whole segment. We calculated coancestry values independently for each segment and then estimated the empirical and theoretical variance (assuming the multinomial model) as described in the Supplementary Material (page 30) of [41], with the simplification that the number of segments is the same for every individual. This is because all individuals have the same genome length (~3,600 cM per haplotype), whereas the total number of shared chunks can vary across individuals. In the case of analysis (A), the *c*-factor value estimated for total genome length was 1.17, compared to 0.39 for the number of chunks.

2.3 Discussion of potential sampling effects

It is possible that sampling strategy may play a role in the population structure we observe, as pointed out in the main text. Here we discuss potential effects. The sampling distribution in our study was determined by the earlier GWAS study [8], as well as the additional filtering we applied (see above). The underlying sampling distribution is therefore likely to reflect the incidences of colorectal cancer cases in different regions (which would reflect the local population density), the involvement rates of hospitals, and the accessibility of cancer patients to the hospitals that provided samples (cases and controls) in the study.

In general, bias in the representativeness of samples from different regions – for example, an excess of rural dwellers, or older people – could potentially have an impact on our ability to detect population structure. Critically, any such biases are likely to be consistent across all regions, since for the vast majority of samples the same sampling strategy was used to recruit participants everywhere. Specifically, around 1400 samples were sourced from the EPICOLON collection in the original GWAS study [8]. Cases were sourced from hospitals and cancer-free controls sourced from the same hospitals and matched on basic demographics, including geographic region of origin. A smaller subset of ~100 samples were sourced from the Spanish National DNA Bank, and also matched to the cases on sex, age, and geographic origin [8].

Sampling density is more likely to be region-specific, because this would have depended on the involvement rates of hospitals in different regions. Inhomogeneity of sampling density might prevent us from observing fine-scale structure where present, so it might be the case that structure is more extensive in some places than we observe. We have taken several steps to mitigate this possibility: excluding familiarly-related samples from the analysis; excluding samples without all four grandparents nearby (80km) when we plot samples on maps, to homogenize this aspect of sampling (see Supplementary Table 6 for how different regions were affected); and in the regions with the strongest observed substructure (Galicia), we sub-sampled and re-analysed to show that the structure remained after down-sampling (details in Note Note 4). Furthermore, there are regions with lower sampling density than in Galicia where we do see structure (e.g Asturias and Castilla-Leon; **Figure 1b**), indicating that our approach has the power to detect this with lower-density sampling, if it is there. There are also examples of the reverse, where sampling density is relatively high, but less structure is seen, e.g. in Cataluna with around 200 samples.

Crucially, our approach would not be able to find structure if it was not there, so any regional sampling biases would not affect our conclusion that we observe fine-scale structure in Galicia. Nor would it affect our other main conclusions; for example regarding admixture events, or that genetic structure in Spain coincides with certain historical events. *A priori*, sampling biases would seem more likely to obscure than produce such correspondences.

Note 3 Map-based data visualisation

In figures showing a map of Spain (e.g. **Figure 1b**) each individual is represented by a point placed at the average coordinate (centroid) of their grandparents' birthplaces (coordinates were derived as described above). Where many individuals have the same coordinate, such as in Barcelona, points have been randomly shifted, by no more than 24 Km, to aid visualisation. To visually represent the discrete assignment of individuals to clusters by fineSTRUCTURE, the members of each cluster are represented using the same colour and symbol. We also coloured the background of the maps using the following smoothing procedure:

- The map is divided up into a fine, regular grid of 3km-wide squares, after projecting longitude/latitude coordinates into the above-mentioned coordinate system.
- Each individual i shown on the map contributes an amount h_{si} to each grid-point s, where h_{si} is computed by evaluating a normal density (mean=0, sd=3.5) at the Euclidian distance between the centre of grid-point s and the coordinate of individual i (in units of 10 Km). This is equivalent, up to a constant scaling factor, to evaluating a symmetric, bivariate Gaussian density with mean (0,0), and equal variance in both directions.
- Weights are reduced by a factor of one quarter for individuals whose location falls further than 200 Km from the nearest individual in its assigned cluster. There were between 1 and 30 such individuals (depending on the level of the tree), and this prevents them from contributing disproportionate amounts of colour to regions of low sampling density.
- The total contribution H_{sk} of cluster k to grid-point s is the sum of the weights of individuals assigned to that cluster.
- Multiple colours (one for each cluster) are applied to each square, with transparency according to the relative contributions of each cluster k.

Parameters for the above procedure, such as grid cell size and variance of the smoothing kernel have been chosen such that the visualization emphasises the geographical localities of genetic clusters without suggesting undue certainty about cluster boundaries.

We visualise continuous variables measured for each individual, such as cluster assignment certainty (**Supplementary Figure 1b**), using a similar spatial smoothing procedure. Specifically, for each grid-point s we compute a weighted average (over all individuals) of the continuous variable, where the weights w_{si} are exactly those as defined in Note 0.

All maps were transformed into the Lambert Azimuthal Equal Area (LAEA) projection centred on Madrid (40.5'N, 3.7' W) before plotting. Plots were drawn using the 'spplot' function contained in the 'sp' package in *R* [9].

Note 4 Investigation into ultra-fine-scale structure in Galicia

Many of the inferred clusters are located in the south-west region of Galicia, but relatively few clusters in the rest of Galicia. This difference can be seen by comparing the two clades labelled ‘Galicia_Pontevedra’ and ‘Galicia_central’ in **Figure 1a**. However, this region of Galicia was sampled more densely than other regions, which would provide greater power to detect structure. In order to test whether sampling density was a strong factor in the excess structure detected within the south-west region of Galicia, we conducted a complementary fineSTRUCTURE analysis after sub-sampling individuals from Galicia. Specifically, we used a subset of individuals such that the number of individuals in the set of clusters located in south-west Galicia (‘Galicia_Pontevedra’) was the same as the number of individuals in the clusters located in inland Galicia (95). We kept all the individuals shown on the maps, and then randomly sampled (evenly) from each of the six clusters within ‘Galicia_Pontevedra’ to make up the remainder (96 total). We then re-computed the coancestry matrix, but with just 1,220 individuals, and ran fineSTRUCTURE in the manner exactly as described above for analysis (A), with one exception. In order to focus the analysis on Galicia only we used the ‘continental force file’ option (-F), where the forced population groups were each of the lower-level clusters not part of the clades labelled ‘Galicia_Pontevedra’ or ‘Galicia_central’ (as in **Figure 1**). This option restricts fineSTRUCTURE to find clusters within the set of individuals *not* in the ‘forced’ population groups (i.e. the Galician clusters). Other individuals are allowed to be donors to the individuals of interest, but do not contribute to the parameter inference or the tree-building step (page 11 of the fineSTRUCTURE software manual). This allowed for the possibility that structure within Galicia might be driven by different relationships with groups outside Galicia, while at the same time focusing the analysis only on the behaviour of the Galician clusters. Results of this analysis are shown in **Supplementary Figure 3**. There is good correspondence with the cluster assignments between the two analyses at the level of the tree that is shown in **Figures 1a** and **3a**, confirming that the excess of fine-scale structure in south-west Galicia is still detectable, even under a more even sampling scheme.

The demographic history of Galicia goes some way to explaining this remarkable sub-structure. Galicia maintained its own cultural identity and language as distinct from the rest of Spain [10], and historically, people in Galicia have tended to inhabit small villages rather than towns [11]. Even today, Galicia remains predominantly rural, with 64% of the population residing in rural areas, compared to 44% outside Galicia[1]; and 57% of Galician residents born in Spain before 1961[2] still reside in the same municipality as they were born, compared to 43% in the rest of Spain (Population

[1] This is based on the total resident population minus those that live in urban municipalities or ‘cities’ in 2016, as defined by the Spanish official statistics agency, including the ‘Greater cities’ of Barcelona and Bilbao. See http://www.ine.es/en/prensa/ua_2017_en.pdf for details.

[2] Most (90%) of the Spanish cohort were over 50 years old at the time of data collection (2000-2008), so would have been born around 1960 or earlier.

and Housing Censuses 2011) [12]. Individuals from Galicia in this data do not have unexpectedly low heterozygosity (data not shown), but studies of marital behaviour over the early-to-mid 20th Century within Galicia have shown that there has been a higher proportion of marriages between uncles/nieces or aunts/nephews and first cousins than in other parts of Spain [13, 14], suggesting that a highly localised marital behaviour is more common in Galicia than elsewhere. Galicia has been the subject of a number of genetic studies, all using small numbers of genetic markers (mtDNA, Y-chromosome, Alu-insertions). While some found significant differences in allele or haplotype frequencies between Galicia and other parts of Spain, such as the Basque region [19] none have been able to detect strong population structure within Galicia [15-18]. Our analysis indicates that there is significant genetic sub-structure within Galicia, especially in the south-west, and which is probably a result of isolation from the rest of Spain, as well as localised societal organisation within Galicia.

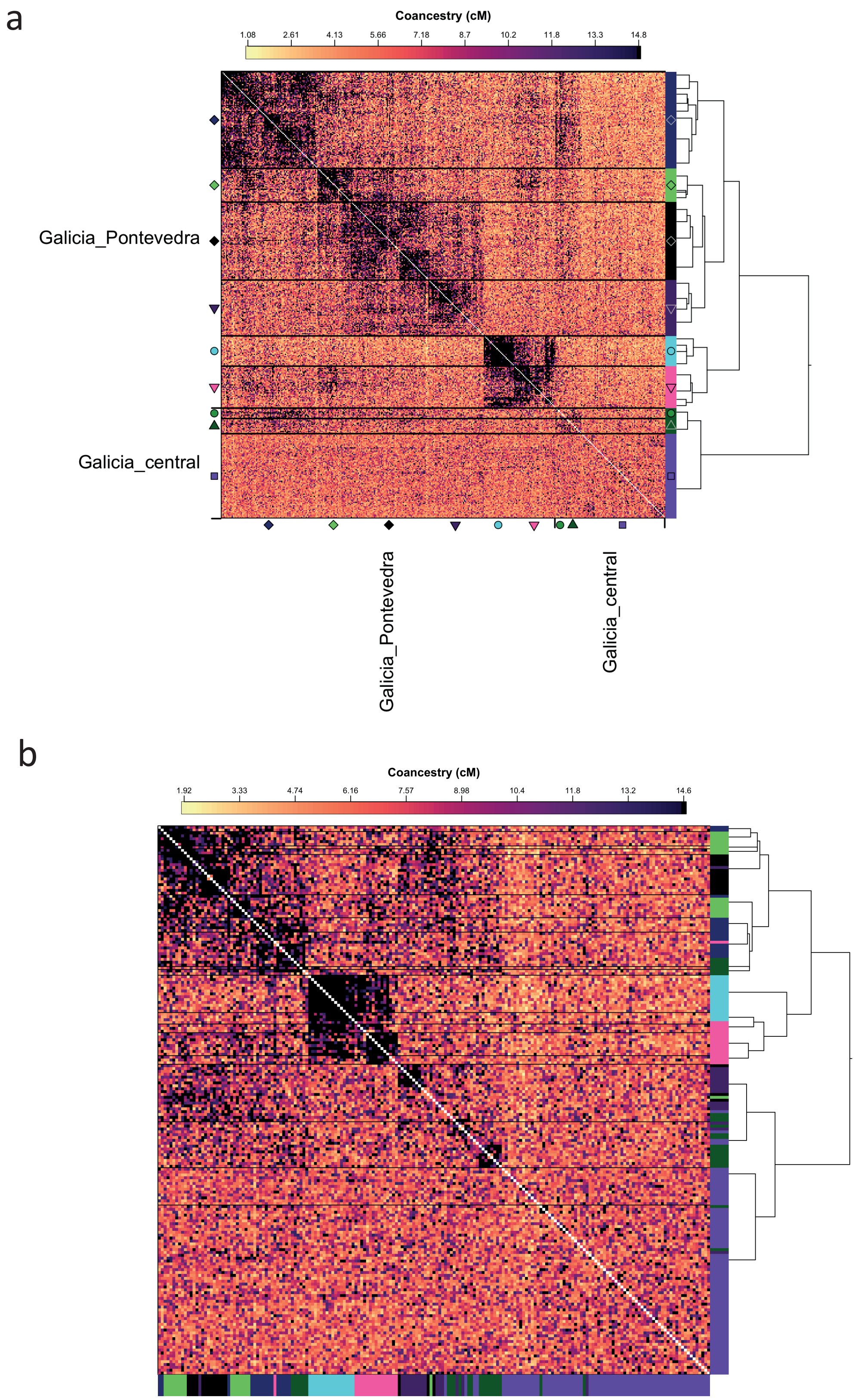

Supplementary Figure 3 | Effect of sub-sampling on fine-scale structure in Galicia. We tested the effect of high density sampling in the region of south-west Galicia by conducting a fineSTRUCTURE analysis on a subset of individuals such that the number of individuals in the set of clusters located in south-west Galicia (labelled in (a) as 'Galicia_Pontevedra') was the same as the number of individuals in the clusters located in other parts of Galicia (details in Supplementary Note 4). **(a)** Section of the coancestry matrix shown in **Figure 4a** that involves the clusters located primarily in Galicia (n=384). **(b)** Coancestry matrix and fineSTRUCTURE tree inferred after sub-sampling (n=191). The colours in the axes indicate which of the clusters each individual belongs to in the original analysis, as shown in (a).

Note 5 Signals of drift and admixture in coancestry matrices

In the main text we report evidence of admixture from Basque-like cluster in the north of Spain with a cluster located to the south (labeled 'central' in **Figure 1**) by using information in the coancestry matrix (**Figure 4a**). Here we present theoretical arguments that support this claim.

In previous work [6] it has been shown that coancestry (as we have used it) closely approximates the amount of genome (in cM) for which an individual *i* shares its most recent common ancestor with another individual *j*, out of all the individuals in the sample. We will assume this holds for our application also. Therefore, patterns in this matrix are informative of coalescence rates within and across clusters inferred by fineSTRUCTURE.

Specifically, excess coancestry between individuals in the same cluster (within-cluster coancestry) is often observed (for examples see **Figure 4b**), and indicates more rapid historical coalescence rates among ancestors of that cluster, than across clusters. This is because individuals within a cluster have similar patterns of shared ancestry, so often share more ancestors. However, it is possible for this not to be the case, and this is informative of admixture, which can also produce recent shared ancestry across clusters. Different possible histories for two populations α and β are shown in **Figure 4d**: either a pure split model followed by a period of isolation without subsequent admixture/gene flow; or a split followed by one or more pulses, or period, of genetic exchange, in one or both directions.

In any pure two-way split model (left-most diagram in **Figure 4d**) any individual in β must always have more coancestry — on average — with another individual in β than they do with another individual in α. This is because at all times in the past, the coalescence rate between ancestors of two members of population β is at least as large as that between the first population β member and a population α member. This is still true even if there is migration purely from the ancestral group of β (labeled β') into that of α (labeled α'), in an otherwise straightforward split (second-left diagram in **Figure 4d**). It follows that if individuals in β have more coancestry with individuals in α than they do with individuals in their own cluster, then the shared history scenario, or one-directional admixture cannot be true, so the admixture scenario (α' → β') must have occurred (right-hand diagrams in **Figure 4d**). This property is sufficient, but not necessary for indicating admixture. In other words, it does not necessarily hold that if α' → β' admixture has occurred then β will show more coancestry with α than with itself. This requires both a high enough level of admixture, and a higher coalescence rate among ancestors of α than those of β.

Note 6 Details of defining non-Spanish donor groups

In order to define a set of 'donor groups' using the combined European, north African, and sub-Saharan African individuals, we applied fineSTRUCTURE as described in Note 2 in several rounds, each using the following sets of individuals:

(CI) All individuals combined (excluding Spanish but including Portuguese)
(CII) Individuals from north Africa only
(CIII) Individuals from Europe only.

In analysis (CI) fineSTRUCTURE cleanly split the 3 main groups corresponding to Europe, north Africa, and sub-Saharan Africa, as well as inferring finer sub-structure. However, in order to maximise the power to detect finer scale structure [6], we obtained fineSTRUCTURE results for the north African and European groups independently. That is, using coancestry matrices that only allow copying within each set of individuals in (CII) and (CIII), respectively. We then defined 29 donor groups based on the clusters and hierarchical trees inferred by fineSTRUCTURE in these three analyses. We considered the following factors in defining donor groups. Ideally, each donor group would contain about the same number of samples, and not be too small. Donor groups should also be relatively homogeneous with respect to their shared ancestry with the population of interest (in this case Iberia), although could be heterogeneous within themselves. We therefore prioritised donor group size over capturing finer scale structure that might exist within donor groups themselves.

For the European samples we used results from analysis (CIII). Some clades of the tree contained clusters with very small numbers of individuals, especially the clade involving mostly Swiss individuals. To avoid using small clusters as donor groups, we chose a level of the hierarchical tree such that all further splits were smaller than 5, and then excluded samples from any group with a size less than or equal to 15. There were 50 such samples. We also excluded an additional 3 samples from France, which we found shared unusually large amounts of coancestry (> 12.5cM) with individuals in the Basque-centred clusters in the Spanish cohort (mean coancestry between the French donor group and Basque-centred clusters was 6.2cM with s.d. 1.1).

For the north African samples we used analysis (CII). Simply removing smaller clusters would have resulted in excluding a significant proportion of samples, so we employed a different strategy. Starting with the smallest cluster, we merged this with the cluster nearest it in the tree, and repeated this process until all resulting clusters were at least size 15.

For the sub-Sahara African samples we used analysis (CI). These individuals formed their own clade of the hierarchical tree, and fineSTRUCTURE identified the three main population groups: Maasai in Kinyawa, Kenya (MKK); Luhya in Webuye, Kenya

(LWK); and Yoruba in Ibadan, Nigeria (YRI). Some sub-structure within these groups was also identified, especially among the MKK individuals. However, since the coancestry sharing between these smaller clusters and north African and European samples is relatively homogeneous, we merged all the clusters within the MKK and LWK sub-clades, resulting in four sub-Saharan African donor groups.

This procedure resulted in a total of 29 donor groups with median size 30, and minimum size 16, totaling 1,386 individuals (excluding the group of 117 Portuguese individuals – see note in Methods). Their locations are shown in **Figure 6a**. Labels of the inferred groups are based on the sampling locations of most of the individuals in a given group. In some cases the majority of individuals were split across two locations, and this is indicated by a multi-region label (e.g. Germany-Hungary).

Note 7 Details of estimating ancestry profiles

7.1 Cluster-based ancestry profiles

We estimated ancestry profiles for each of the Iberian clusters using the procedure previously described in [7]. Specifically, we use CHROMOPAINTER to compute a coancestry vector for each Iberian individual, where we only allow them to copy from haplotypes in the donor groups. We sum the elements of these vectors corresponding to individuals from each donor group, thus forming a vector for each Iberian individual where each element corresponds to a donor group. We then average over individuals in each Iberian cluster to form a single coancestry vector for each cluster and normalise so these vectors sum to one. Equivalent vectors are also computed for each of the donor groups, but allowing them to copy from other individuals within their own group. We computed all these vectors using a leave-one-out procedure. That is, since individuals cannot copy from themselves we excluded one individual (sampled randomly) from each of the donor groups, except the group being painted, so that the number of possible donors from each group is always the same. Using these cluster- and group-averaged copying vectors we find a smaller set of donor groups which together (as a linear mixture) can best account for the raw coancestry vector of each Iberian cluster. That is, we solve (for $\overline{x}_i$) the non-negative linear least squares problem,

$$\overline{y}_i = \overline{x}_i D + e \quad \text{such that} \quad \overline{x}_i \geq 0 \qquad (2)$$

where $\overline{y}_i$ is group-averaged coancestry vector for Iberian cluster *i*, and D is the matrix of group-averaged coancestry vectors of each donor group. The vector of coefficients, $\overline{x}_i$, is the ancestry profile for cluster *i*, and its elements sum to 1. Results for six Iberian clusters are shown in **Figure 6b**. We also did a complementary analysis where we treated each *donor group* in turn as $\overline{y}_i$ (the results are shown in **Supplementary Figure 4**). To do this we first excluded the elements of $\overline{y}_i$ and D that correspond to coancestry within its own group, and re-normalised the coancestry vectors to sum to 1.

We measured uncertainty in the ancestry profiles by re-estimating $\overline{x}_i$ using the coancestry vectors for a set of pseudo individuals. Each pseudo individual is formed by randomly selecting an individual in cluster *i* for each chromosome, and summing the observed chromosome-level coancestry vectors across all chromosomes. We then compute 1000 such re-estimations and report the range of the inner 95% of the resulting bootstrap distribution.

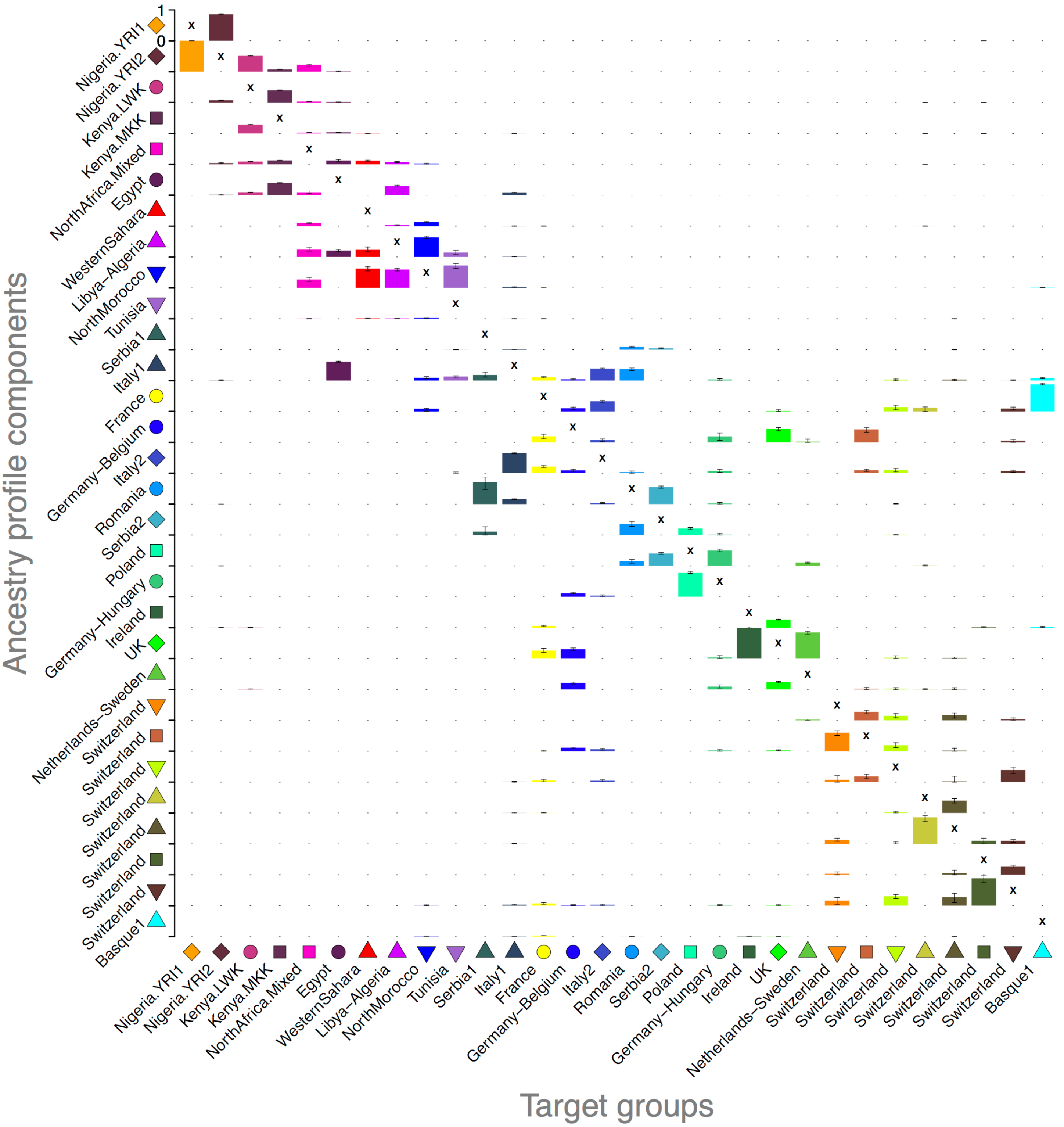

Supplementary Figure 4 | Ancestry profiles of non-Iberian groups and Basque cluster. (a) Each column shows the estimated ancestry profile (Methods) for each of the non-Iberian donor groups plus a Basque cluster (labelled 'Basque1' in **Figure 1a;** 60 individuals). The groups are ordered based on the fineSTRUCTURE analyses we used to define the donor groups (Methods) and labelled based on the locations of most individuals in each group (see **Figure 6a** for locations and group sizes). The heights of the bars within each column sum to one. Error bars show the range of the inner 95% of 1,000 bootstrap resamples (Methods) and are only shown if the point estimate was greater than zero. Within-group copying was not allowed under the model, indicated by an X on the diagonal entries.

7.2 Spatially smoothed ancestry profiles

The availability of fine-scale geographic information for many of the Spanish individuals allowed us to estimate the spatial distribution of shared ancestry (**Figures 5c** and **5d**). Instead of averaging coancestry over individuals within a cluster, we average across geographic space using the following kernel smoothing method.
Given a set of N coancestry vectors $\overline{y}_i$ (one for each individual *i*), we computed a new coancestry vector $\overline{y}_s$ for each grid-point s in a fine grid across Spain such that,

$$\overline{y}_s = \frac{\sum_{i=1}^{N}(w_{si}\overline{y}_i)}{\sum_{i=1}^{N} w_{si}} \tag{3}$$

where w_{si} is the two-dimensional, zero-centred symmetric Gaussian function evaluated at d_{si}, the Euclidean distance between the centre of grid-point s and the coordinate of individual i (in units of 10 Km). That is,
We allow the parameter σ_s^2 to vary to reflect the amount of real data available around each grid-point. Specifically, each σ_s^2 is set such that the sum of the weights w_{si} is the same for all grid-points. That is, for each grid-point s we solve,

$$\sum_{i=1}^{N} e^{-\frac{d_{si}^2}{2\sigma_s^2}} = k \tag{4}$$

$$w_{si} = e^{-\frac{d_{si}^2}{2\sigma_s^2}} \tag{5}$$

for σ_s^2, where k is set to the maximum value (over all grid-points) of the left-hand-side when using $\sigma_s^2 = 3.5$. In practice we solve this equation numerically for each grid-point using an implementation of Nelder and Mead's simplex algorithm implemented in *R* ('optim' function) [19]. This has the effect that all grid-points have $\sigma_s^2 \geq 3.5$ and grid-points with a lower density of nearby data points have weights w_{si} spread more thinly over larger distances. This ensures, for example, that a single isolated data point will not contribute heavily to the coancestry vectors of nearby grid-points.

We then compute ancestry profiles for each of the grid-points in the same way as for the Iberian clusters (described above), but setting $\overline{y}_i = \overline{y}_s$ instead of the cluster-averaged coancestry vector. We visualize the results by colouring each grid point according to the value of its coefficient for a single donor population of interest (e.g NorthMorocco in **Figure 5c**). For any grid-point s and individual i located within the borders of Portugal we set w_{si} to 1, because we have no fine-scale geographic information for these individuals. This means in Portugal there is just one colour, based on the mean coancestry vector across individuals located in the region. Results are visualised in **Supplementary Figure 5**.

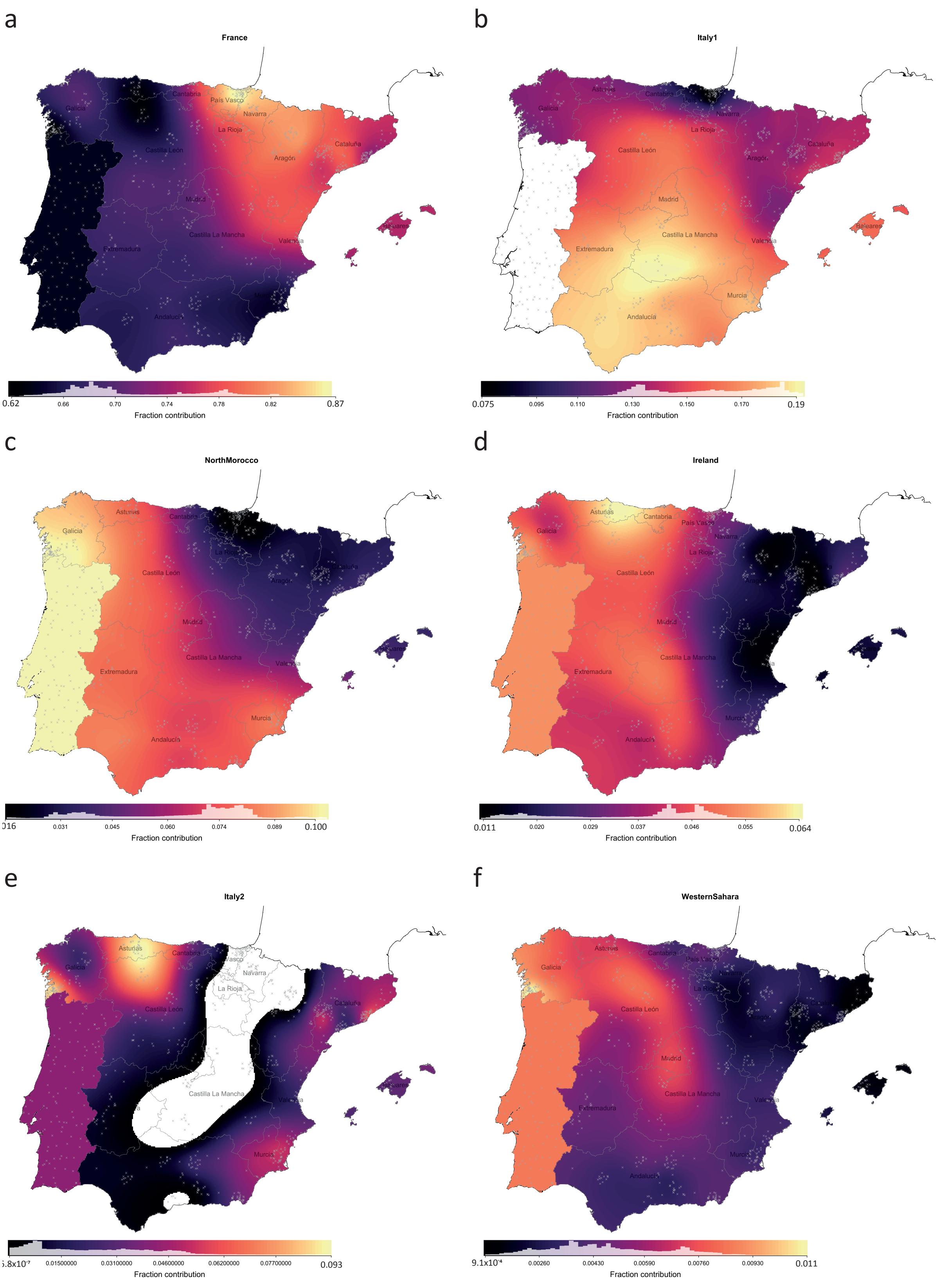

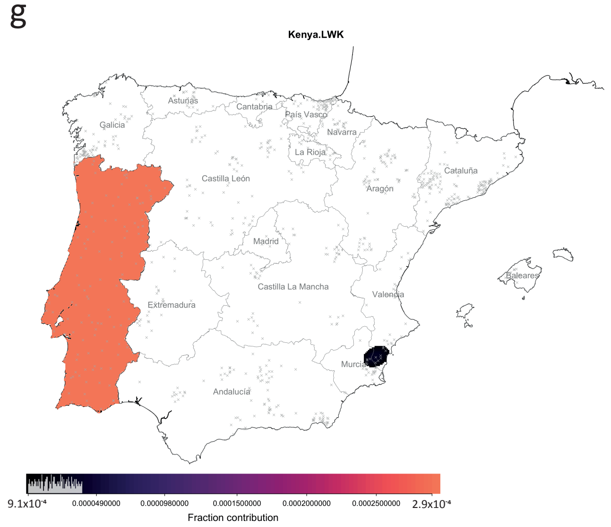

Supplementary Figure 5 | Components of spatially-smoothed ancestry profiles for main genetic contributors to Iberia. A spatially-smoothed ancestry profile has been computed for each point on a spatial grid across Spain (Methods). Each map shows the fraction contributions from the stated donor group (e.g. 'NorthMorocco'). These seven groups are exactly the same contributors to the cluster-based ancestry profiles shown in **Figure 6b**). Note the colour scale changes because the maximum contribution differs across donor groups. White areas indicate where the donor group contributed zero to the ancestry profile. The histogram on the scale bars shows the distribution of values across grid-points on the map (excluding zero).

7.3 Discussion of ancestry profile results

Here we discuss the ancestry profiles for the six Iberian clusters in more detail (referring to **Figure 6b**). Only seven of the 29 donor groups (six with >1% contribution to any group) show any meaningful contribution in ancestry profiles, and are primarily in Western and Southern Europe, and north-west Africa. For all six Iberian clusters the largest contribution comes from France, with smaller inferred contributions that relate to present-day Italian and Irish samples. Because these contributions are present and dominate overall ancestry throughout Spain, they might represent ancient ancestry components, rather than recent migration. The remaining contributions come from North Morocco and Western Sahara, and show strong (and highly significant) regional differences, varying continuously across Spain. The North Moroccan component steadily declines from 11% in the far west to near 0% in the Basque region. The pattern of North Moroccan contributions is similar to the Western Saharan contributions, and approximately opposite to the pattern of French contributions. Regional variation is less marked for the contributions from Irish and Italian donor groups, perhaps implying these relate to non-identical ancestral populations to that of the French group. A very small (0.2%) but statistically non-zero contribution from sub-Saharan Africa is present for just one cluster (red triangles), which contains individuals largely from Portugal and regions of southern Iberia, such as Andalucia, and Murcia. This group otherwise has a similar profile to the west-most cluster (yellow squares), indicating that this cluster is differentiated only by an excess of ancestry sharing with individuals from sub-Saharan Africa. Furthermore, in general for Iberia there is a strong linear correlation between north African and sub-Saharan ancestry sharing (coancestry), but for many individuals in this group there is a larger sub-Saharan African component than would be expected given their north African component (**Supplementary Figure 6a**).

Notably, the Basque-centred cluster has a markedly different profile from the rest. Firstly, it has much lower, or zero contributions from donor groups that contribute to all other clusters: Italy, NorthMorocco, and WesternSahara, and a very large contribution of 91% (88-93) from France. Additionally, the model fit for this cluster is strikingly less good than that for the other clusters (**Supplementary Figure 6b**), suggesting that Basque-like DNA is less well captured by the mixture of donor groups in this data set. Specifically the Basque share even more DNA with the French group than predicted by their mixture representation, which might reflect, for example, that the DNA the Basque share with present-day French is only a subset of modern French ancestry. This pattern is seen for other Spanish groups also, but to a much lesser extent.

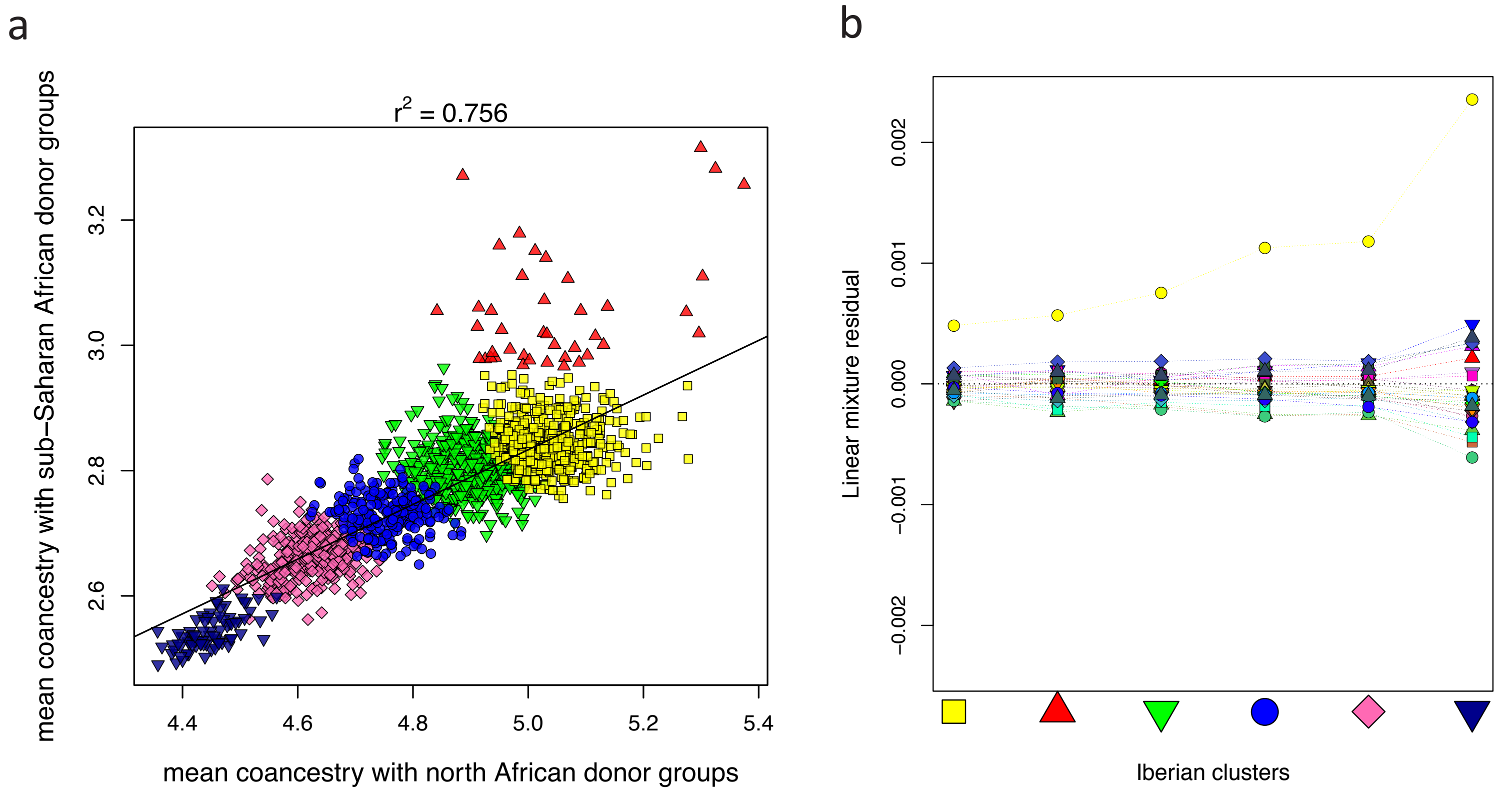

Supplementary Figure 6 | Properties of cluster-based ancestry profiles. (c) Correlation (Pearson's r^2) in Iberians' ancestry sharing with north African and sub-Saharan African donor groups. Each point represents an Iberian individual (n=1,530) with colours and symbols corresponding to the Iberian clusters shown in **Figure 5a**. The *x* and *y*-axes show the mean coancestry with north African and sub-Saharan African individuals, respectively. We defined 'sub-Saharan African' as donor groups Kenya.LWK, Kenya.MKK, Nigeria.YRI1 and Nigeria.YRI2; 'north African' as the donor groups NorthAfrica.Mixed, WesternSahara, NorthMorocco, Tunisia, Libya-Algeria, and Egypt. **(d)** Residuals for each component of the Iberian ancestry profiles shown in **Figure 6b**. Each point represents the residual for a donor group indicated by a colour/symbol. Positive values on the y-axis indicate that the observed coancestry component is larger than the fitted component.

	■ Galicia-Portugal (407)	▲ Portugal-Andalucia (41)	▼ west (421)	● central (240)	◆ Aragon-Cataluna (344)	▼ Basque (77)
**France**	0.6416 (0.6291 - 0.6566)	0.6296 (0.5838 - 0.6661)	0.6749 (0.6615 - 0.6895)	0.7001 (0.68 - 0.718)	0.7835 (0.7673 - 0.7984)	0.9089 (0.8838 - 0.9289)
**Italy1**	0.1501 (0.1422 - 0.1584)	0.1717 (0.1436 - 0.1979)	0.1665 (0.158 - 0.1748)	0.1589 (0.1485 - 0.1695)	0.1317 (0.1231 - 0.1405)	0.0477 (0.0347 - 0.0602)
**NorthMorocco**	0.1092 (0.1065 - 0.1117)	0.1006 (0.0837 - 0.1138)	0.0834 (0.0799 - 0.0858)	0.0596 (0.0568 - 0.0621)	0.0294 (0.0274 - 0.0314)	0 (0 - 0)
**Ireland**	0.0467 (0.0415 - 0.0511)	0.0398 (0.0252 - 0.0542)	0.0372 (0.0322 - 0.0419)	0.0392 (0.0331 - 0.0456)	0.0214 (0.0161 - 0.0271)	0.042 (0.0312 - 0.0554)
**Italy2**	0.0423 (0.0273 - 0.0558)	0.022 (0 - 0.0656)	0.03 (0.0149 - 0.0447)	0.0401 (0.0206 - 0.0598)	0.0308 (0.0152 - 0.0474)	0 (0 - 0.0157)
**WesternSahara**	0.0099 (0.0085 - 0.0112)	0.0068 (0.0025 - 0.0111)	0.0061 (0.005 - 0.0071)	0.0021 (0.0008 - 0.0033)	0.0029 (0.002 - 0.0038)	0.0012 (0.0001 - 0.0023)
**Kenya.LWK**	0 (0 - 0)	0.0018 (0.0005 - 0.0029)	0 (0 - 0)	0 (0 - 0)	0 (0 - 0)	0 (0 - 0)

Supplementary Table 1: Point estimates and confidence intervals for ancestry profiles of Iberian clusters as visualised in Figure 6b. Each column is an Iberian cluster (number of samples in parentheses), and each row is a donor group. See Note 7.1 for details of this analysis.

Note 8 Details of GLOBETROTTER analyses

8.1 Details of the two analyses

We conducted two analyses using GLOBETROTTER. The first (gtA) was designed to detect admixture event(s) in the history of Iberia that might involve any combination of non-Iberian source populations, without any prior assumptions on the nature of the event. The second analysis (gtB) was designed to detect only admixture event(s) involving a Basque-like source population, i.e. based on a prior hypothesis. In each case we defined a set of target groups within which to look for an admixture event; a set of donor groups, which we allow to be donors in the initial 'painting'; and a set of surrogate populations, which we allowed GLOBETROTTER to consider as components of any admixture event. These details are summarised in Supplementary Table 2. In order to speed up computation time, in both analyses we restricted the number of individuals within a target group to 100 by randomly sampling groups with more than 100 individuals. See Supplementary Table 3 for resulting sample sizes in each target group. We otherwise ran GLOBETROTTER as recommended by the authors (GLOBETROTTER Instruction Manual [20]). In all figures and tables relating to GLOBETROTTER results we report the results using the 'null.ind: 1' procedure. That is, the coancestry curves are normalised by 'across-individual' coancestry curves, which are constructed exactly as described above, but where only pairs of chunks in the haplotypes of *different individuals* within the target population are compared. In practice we found the results were similar to the 'null.ind: 0' versions, but are less likely to be influenced by any bottleneck effects since an admixture event [20].

Analysis	Target groups	Donor groups	Allowed surrogate groups
**gtA**	6 Iberian clusters, inferred on the basis of haplotype sharing with non-Iberians only. These are shown in Figure 5a.	29 donor populations defined as discussed above	29 donor populations defined using fineSTRUCTURE (Note 6)
**gtB**	Portuguese cluster<br><br>20 Spanish clusters from fineSTRUCTURE analysis (A), excluding 60 individuals in the clade labelled 'Basque1' (Figure 1a).<br><br>Individuals within the clade labelled 'Galicia_Pontevedra' were combined into for this analysis.	Basque1<br><br>29 donor populations defined using fineSTRUCTURE (Note 6)	Basque1<br><br>A subset of donor groups:<br>Germany-Belgium<br>Germany-Hungary<br>Netherlands-Sweden<br>Poland<br>Romania<br>2 x Serbia<br>7 x Switzerland<br>UK

Supplementary Table 2 | The target, copying and surrogate groups that we defined in two GLOBETROTTER analyses. Labels of target and surrogate groups for analysis gtB refer to those shown in **Figure 1a** (Spanish clusters) and **Figure 6a** (non-Iberian groups).

(a)

Target population	Sample size	One date (generations)	One date (year)	Two dates date 1 (generations)	Two dates date 1 (year)	Two dates date 2 (generations)	Two dates date 2 (year)	fit.quality.1event	maxR2fit.1date	maxScore.2events
▼ Basque	100	40 (36-45)	861 (706-973)	N/A	N/A	N/A	N/A	0.992	0.978	0.186
◆ Aragon-Cataluna	100	39 (34-44)	869 (732-1018)	13 (3-35)	1591 (984-1873)	57 (45-103)	365 (922BC-714)	0.993	0.976	0.515
● central	100	36 (31-40)	957 (855-1088)	21 (15-29)	1374 (1164-1558)	67 (48-88)	86 (502BC-612)	0.996	0.978	0.481
▼ west	100	39 (37-43)	864 (760-942)	N/A	N/A	N/A	N/A	0.990	0.980	0.237
▲ Portugal-Andalucia	41	30 (23-37)	1119 (923-1336)	11 (7-24)	1647 (1300-1774)	45 (50-74)	696 (104BC-567)	0.996	0.962	0.564
■ Galicia-Portugal	100	37 (32-42)	929 (802-1085)	10 (3-35)	1698 (984-1873)	49 (45-103)	589 (922BC-714)	0.989	0.978	0.616

(b)

Target population	Sample size	One date (generations)	One date (year)	Two dates date 1 (generations)	Two dates date 1 (year)	Two dates date 2 (generations)	Two dates date 2 (year)	fit.quality.1event	maxR2fit.1date	maxScore.2events
◆ Basque2	26	21 (12-33)	1380 (1047-1639)	N/A	N/A	N/A	N/A	1.000	0.520	0.019
▼ Basque2	21	21 (15-29)	1375 (1162-1544)	N/A	N/A	N/A	N/A	1.000	0.597	0.030
▼ northCentral	52	23 (17-30)	1319 (1140-1486)	N/A	N/A	N/A	N/A	1.000	0.811	0.102
○ LaRioja	23	16 (11-23)	1514 (1332-1670)	N/A	N/A	N/A	N/A	1.000	0.677	0.013
◇ SouthCoast	13	21 (16-26)	1373 (1241-1507)	N/A	N/A	N/A	N/A	1.000	0.467	0.010
△ central	100	25 (21-28)	1276 (1183-1387)	N/A	N/A	N/A	N/A	1.000	0.894	0.003
■ Murcia	15	24 (16-32)	1304 (1061-1525)	N/A	N/A	N/A	N/A	1.000	0.468	0.008
▲ Aragon-Valencia	29	19 (14-24)	1431 (1283-1568)	N/A	N/A	N/A	N/A	1.000	0.691	0.013
● Aragon-Valencia	100	27 (22-31)	1202 (1087-1345)	N/A	N/A	N/A	N/A	1.000	0.838	0.037
□ Valencia	27	25 (16-33)	1273 (1035-1515)	N/A	N/A	N/A	N/A	1.000	0.593	0.012
● Cataluna	24	25 (16-33)	1272 (1039-1511)	N/A	N/A	N/A	N/A	1.000	0.602	0.003
■ Cataluna	100	25 (21-29)	1281 (1167-1371)	N/A	N/A	N/A	N/A	1.000	0.889	0.016
▼ Baleares	13	19 (12-29)	1447 (1151-1636)	N/A	N/A	N/A	N/A	0.999	0.385	0.014
■ west	100	24 (20-28)	1299 (1195-1406)	N/A	N/A	N/A	N/A	1.000	0.863	0.012
▲ west	100	24 (20-30)	1285 (1132-1398)	N/A	N/A	N/A	N/A	1.000	0.834	0.036
● Asturias	47	24 (19-29)	1303 (1156-1445)	N/A	N/A	N/A	N/A	1.000	0.763	0.008
■ Galicia_central	100	20 (16-24)	1394 (1282-1517)	N/A	N/A	N/A	N/A	1.000	0.842	0.017
▲ Galicia_central	13	28 (14-42)	1190 (786-1572)	N/A	N/A	N/A	N/A	1.000	0.275	0.027
● Galicia_central	9	25 (12-50)	1272 (570-1637)	N/A	N/A	N/A	N/A	1.000	0.310	0.012
◆ Galicia_Pontevedra	100	22 (19-26)	1340 (1245-1447)	N/A	N/A	N/A	N/A	1.000	0.892	0.010
● Portugal	100	24 (21-29)	1300 (1149-1391)	N/A	N/A	N/A	N/A	1.000	0.849	0.038

Supplementary Table 3: Results of two GLOBETROTTER analyses. Details of each analysis are outlined above, and Supplementary Table 1 shows how each of the target groups (rows) were defined. Unless otherwise stated, date estimates are reported in years CE and based on a generation time of 28 years and a 'now' date of 1940 (the approximate average birth-year of this cohort) 'present' date of 1940. Dates represented in generations ago have been rounded to the nearest generation. Date estimates for a two-date event are only shown for target populations where 'maxScore.2events' > 0.35. **(a)** Results for analysis gtA. The coloured symbol of each target group corresponds to an Iberian cluster as shown in **Figure 5a**. **(b)** Results for analysis gtB. The coloured symbol of each target group corresponds to Spanish clusters as shown in **Figure 1**, plus Portugal. Note that the clade labelled 'Galicia_central was combined into one target group for this analysis.

Target population	Kenya.LWK	Egypt	Kenya.MKK	Tunisia	Libya-Algeria	Nigeria.YRI	Western-Sahara	Total fraction (minor)	France	Italy2	Ireland	UK	Italy1	North-Morocco	Tunisia	Western-Sahara	Total fraction (major)
▼ Portugal-Andalucia	0.023	0.000	0.009	0.000	0.000	0.540	0.428	0.04	0.601	0.084	0.056	0.000	0.210	0.035	0.008	0.006	0.96
◇ Aragon-Cataluna	0.000	0.000	0.000	0.060	0.324	0.228	0.388	0.09	0.553	0.034	0.182	0.232	0.000	0.000	0.000	0.000	0.91
● west	0.000	0.000	0.000	0.054	0.083	0.196	0.667	0.10	0.385	0.294	0.189	0.132	0.000	0.000	0.000	0.000	0.90
▽ Basque	0.000	0.000	0.098	0.063	0.000	0.245	0.594	0.07	0.490	0.000	0.222	0.288	0.000	0.000	0.000	0.000	0.93
▲ Galicia-Portugal	0.000	0.012	0.043	0.051	0.000	0.248	0.645	0.08	0.474	0.363	0.156	0.000	0.008	0.000	0.000	0.000	0.92
□ central	0.000	0.020	0.145	0.000	0.000	0.319	0.516	0.05	0.521	0.315	0.160	0.000	0.000	0.000	0.005	0.000	0.95

Supplementary Table 4: Estimated admixture proportions for GLOBETEROTTER analysis gtA. These values are visualized on the left-hand side of **Figure 5b** in the main text.

8.2 Evaluating the statistical support for inferred admixture events and different scenarios

As recommended by the authors of GLOBETROTTER [20] we first tested for evidence of any admixture within each target population by running 100 dating bootstraps using the 'null' procedure under both a one-date and two-date admixture scenario. If D is the number of one-date bootstraps where the inferred date is either greater than 400 or less than 1, then the *p*-value for any admixture is D/101. A *p*-value less than 0.01 indicates evidence of admixture. In all target groups for both analyses, all bootstrap date estimates were within this range, implying evidence of an admixture event, or events, for every target group (Supplementary Table 3).

After identifying the presence of admixture, we next evaluated evidence for more complex admixture events (e.g. multiple dates or more than two source populations). GLOBETROTTER automatically tests for these using a series of criteria based on how well the coancestry curves fit the models for different types of admixture scenarios [20]. Using GLOBETROTTER's automated criteria, for all target populations there was only evidence for one-date or two-date admixture events. However, given the potentially complex nature of admixture in Iberia, we departed slightly from GLOBETROTTER's automatic criteria when evaluating the evidence for one-date verses two-date admixture events. Specifically, evidence of a two-date admixture scenario can be assessed by measuring how much additional variance is explained by fitting the coancestry curves to a two-date model compared to a one-date model (see 'maxScore.2events' in Supplementary Table 3). That is,

$$M = \frac{R^2_{2.date} - R^2_{1.date}}{1 - R^2_{1.date}} \quad (6)$$

where $R^2_{1.date}$ and $R^2_{2.date}$ is the goodness-of-fit (coefficient of determination) for a coancestry curve fitted under one-date and multiple-date admixture model, respectively. Under GLOBETROTTER's automatic criteria, if the maximum value of *M* across all inferred coancestry curves ('maxScore.2events' in Supplementary Table 3) is greater than 0.35 this is considered evidence for multiple-date admixture (a value determined by simulations [20]). In our analysis we considered *M* separately for each pair of surrogate groups inferred to be part of the modern-day mixture representing the admixing sources. This revealed further complexity in our data.

In the case of analysis gtB, there was no evidence that a two-date admixture model fitted better than a one-date model (the maximum *M* for any pair of surrogate populations was 0.1). In the case of analysis gtA, the coancestry curves fit a one-date admixture model very well (across all target groups and coancestry curves the $R^2_{1.date}$ has mean 0.92 and standard deviation 0.1). However, there was some evidence that a two-date admixture model fit better than a one-date model in 4 out of 6 of the target groups, with the strongest evidence for the group 'Portugal-Andalucia' (see **Supplementary Figure 7**). In these cases, *only* the coancestry curves involving a sub-Saharan African surrogate group fit better to a two-date admixture event. The improved fit for the curve for the sub-Saharan African surrogate group 'Nigeria.YRI1' is visually apparent in the coancestry curve shown in **Supplementary**

Figure 7. We therefore consider the one-date admixture event to be a better fit overall, but that there is some evidence for a second event involving sub-Saharan African-like DNA mixing with European-like DNA. In the target groups where there is evidence of this, GLOBETROTTER infers dates in the range 1370 - 1700 CE (assuming a 28-year generation time).

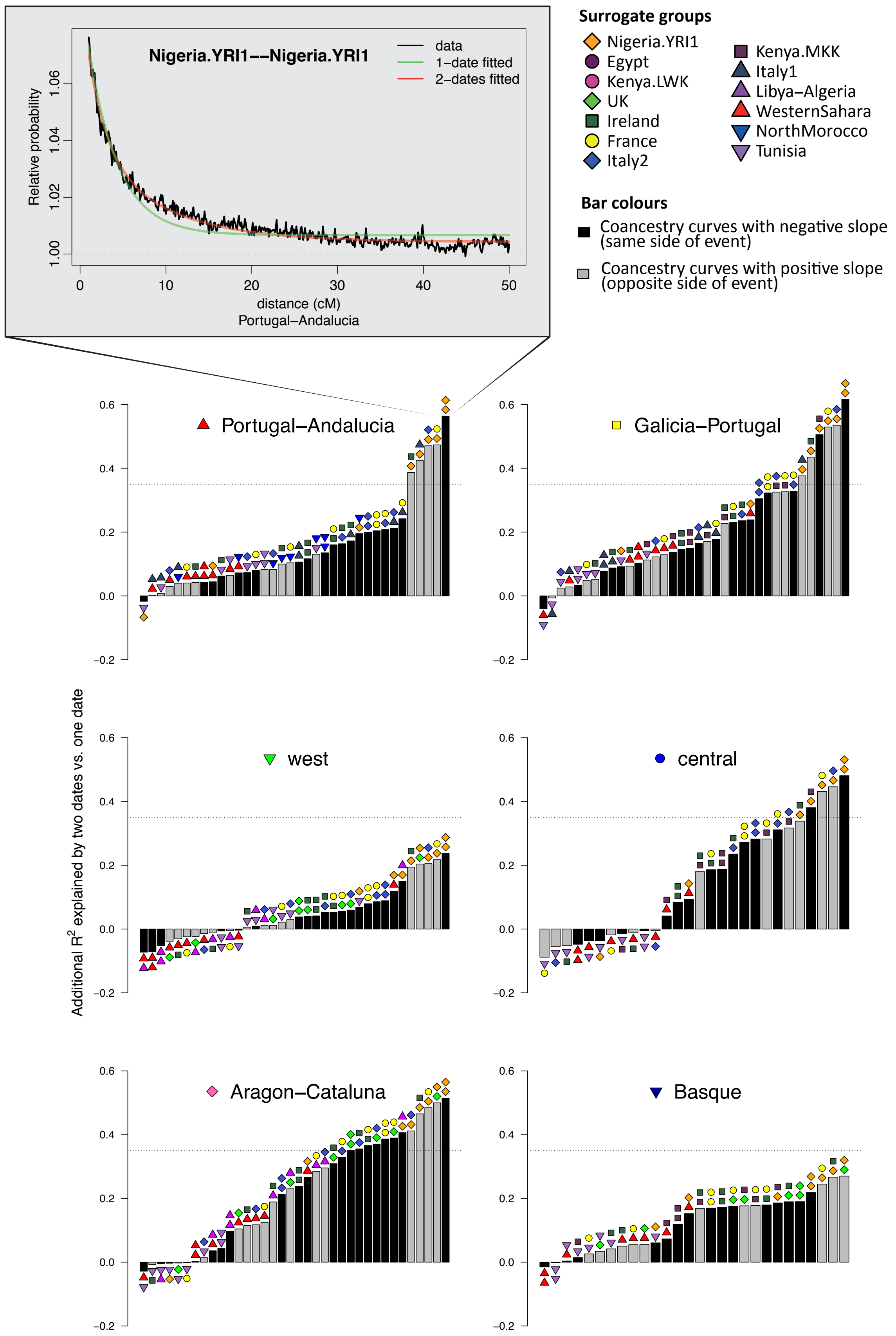

Supplementary Figure 7 | GLOBETROTTER model fit statistics for one-date verses two-date admixture events for each Iberian cluster. All plots refer to results for GLOBETROTTER analysis gtA, allowing all donor groups to be surrogates (Methods; Supplementary Table 2). Barplots for each target Iberian group show the fraction of additional R^2 explained by a two-date admixture model compared to a one-date model (Methods). Negative values can occur when the R^2 for a two-date model is lower than for a one-date model, and the dotted line (0.35) is the value above which there is evidence for a two-date admixture event, as recommended by the authors of GLOBETROTTER. Pairs of surrogate groups are indicated by colors/symbols above the bars; the color of the bars indicates which pairs have a coancestry curve with a negative (black) or positive (grey) slope, which indicate pairs on the same and opposite side of an admixture event, respectively. The inset curve shows the coancestry curves for the target group 'Portugal-Andalucia' for a sub-Saharan African-like surrogate group (YRI), and the fits for one-date and two-dates admixture models.

Note 9 Testing association of historical linguistic regions with genetic clusters

We wished to test whether the linguistic regions in 1300CE explains some variation in our inferred genetic clusters over and above that which can be explained by the boundaries of modern-day autonomous communities and/or the distance between samples. To do this we performed a logistic regression under the model,

$$\ln\left(\frac{p}{1-p}\right) = \mu + d\boldsymbol{D} + a\boldsymbol{A} + l\boldsymbol{L} \tag{7}$$

where,
p : probability of a pair of individuals belonging to the same genetic cluster
$\boldsymbol{D}$: Euclidean distance (in Km) between a pair of individuals
$\boldsymbol{A}$: indicator of whether a pair of individuals are in the same autonomous community.
$\boldsymbol{L}$: indicator of whether a pair of individuals are in the same linguistic region.
μ, d, a, l : the fitted coefficients

We fitted this model using the 14 genetic clusters as shown with background colours in **Figure 1b**. The resulting Z-scores for each coefficient may show inflation due to non-independence between sample pairs, and spatial correlation of linguistic groups. To obtain p-values for the Z-scores relating to l, we therefore used a simulation approach. We generated *K* random partitions of Spain into simulated linguistic regions, each comprising seven polygons (details below). For each partition we constructed a new indicator variable $\dot{\boldsymbol{L}}$, for whether a pair of samples is located in the same polygon and computed the Z-scores for this variable (i.e. coefficient l) after replacing $\boldsymbol{L}$ with $\dot{\boldsymbol{L}}$ in the above model. The appropriate *p*-value for the effect of the true linguistic regions is then *(1+z')/(1+K)*, where *z'* is the number of random partitions with a Z-score greater than the Z-score for $\boldsymbol{L}$. This test is 1-sided because it is only sensible to test whether individuals in the same linguistic region are *more* likely to be in the same cluster.

We performed similar simulations for each of the autonomous community variable ($\boldsymbol{A}$) and distance between samples ($\boldsymbol{D}$). That is, fitting the model after replacing the observed data with simulated data for the variable being tested only. For autonomous community we simulated using the same scheme as described above, but using 16 random polygons (instead of seven) to correspond to the 16 autonomous communities containing samples in our study. For distance between samples we re-calculated the distances after randomly permuting the locations of the samples (but without altering $\boldsymbol{A}$ and $\boldsymbol{L}$).

Using *K*=1000 we estimated $p = 9.99\text{x}10^{-4}$, $p = 0.122$, $p = 7.99\text{x}10^{-3}$ for the effects of distance (d), autonomous community (a), and linguistic region (l) respectively (**Supplementary Figure 8c**). Supplementary Table 5 shows results for association between linguistic region and genetic clusters at different levels of the hierarchical tree (2 – 49 clusters).

All Z-scores were computed using the 'speedglm' function in *R* using a logit link function. Random partitions of Spain were generated by taking the Voronoi partition of seven (or 16 in the case of $\boldsymbol{A}$) randomly sampled locations ('seed points') within the boundaries of Iberia. The Voronoi partition of a set of seeds consists of regions that contain all points closer to each seed than any other seed. We found the Voronoi partitions using the function 'voronoi.polygon' in the *R* package *deldir.* See **Supplementary Figure 8b** for representative examples of random spatial partitions generated in this way.

The above formulation allows us to analyse these variables jointly and include distance between samples in the model. A simpler 2-way test (which does not account for the other covariates, so only assesses marginal significance) for association between linguistic regions and genetic clusters is the chi-squared statistic on the 14x7 contingency table of counts of individuals in each cluster and linguistic region (or 14x16 in the case of autonomous community). Using the same scheme as above to sample random partitions of Spain, both autonomous community and linguistic region yield significant *p*-values of 9.99×10^{-4} and 1.19×10^{-2}, respectively. The difference relative to the previous tests is explained by the inclusion of physical distance in these, but linguistic region remains significant in both types of test.

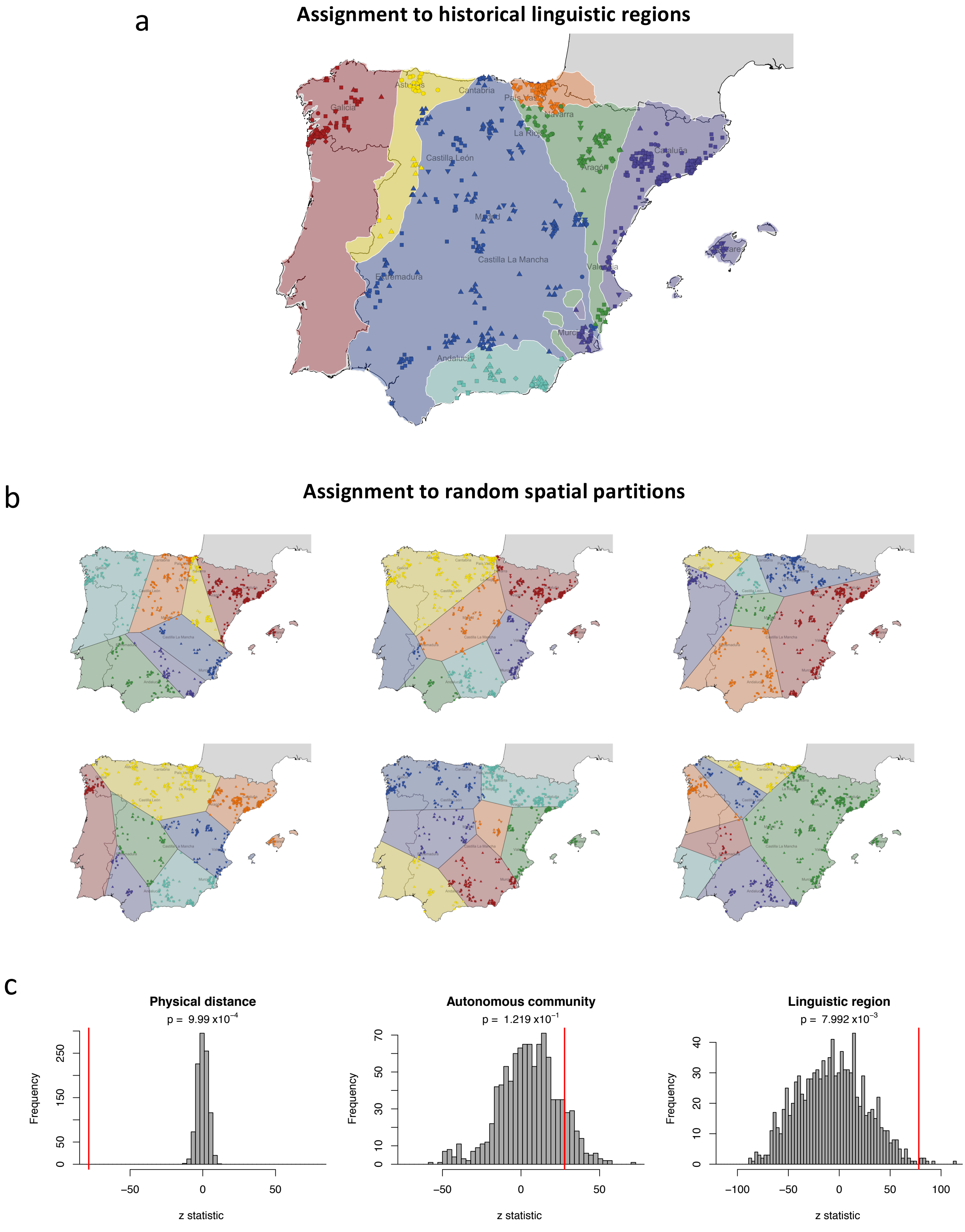

Supplementary Figure 8 | Testing for significance of association between genetic clusters and three predictors. (a) Linguistic regions from 1300CE (as in **Figure 1c**) overlaid with a modern-day map of Iberia showing locations of samples in this study. The linguistic map (a scanned image) was combined with the modern-day map by aligning the shapes of the coastlines, and the points are coloured according to the linguistic region in which the individuals are located. **(b)** Examples of random partitions of Iberia into seven polygons generated from the Voronoi partition of seven randomly-sampled points (Methods). **(c)** Distribution of Z-scores for association with genetic clusters. The histograms show 1000 simulations under a null model (see Note 9 for details) and the vertical red lines show the Z-scores based on the observed data.

Tree level (number of clusters)	*p*-value (linguistic region)	Tree level (number of clusters)	*p*-value (linguistic region)
2	0.20879	26	0.00899
3	0.02697	27	0.00899
4	0.02697	28	0.00899
5	0.01698	29	0.00899
6	0.01698	30	0.00899
7	0.04595	31	0.00899
8	0.04096	32	0.00899
9	0.02498	33	0.00899
10	0.02498	34	0.00899
11	0.02498	35	0.00999
12	0.02498	36	0.00999
13	0.02597	37	0.01099
**14**	**0.00799**	38	0.01099
15	0.00799	39	0.01099
16	0.00799	40	0.01099
17	0.00899	41	0.01099
18	0.00899	42	0.01099
19	0.00699	43	0.01099
20	0.003	44	0.01099
21	0.003	45	0.01598
22	0.003	46	0.01598
23	0.003	47	0.01598
24	0.003	48	0.01598
25	0.003	**49**	**0.00599**

Supplementary Table 5 | Evidence for association of linguistic regions with genetic clusters for different levels of the hierarchical tree. Those in bold are the two levels of the tree illustrated in **Figure 1b**, although note that for ease of visualisation in **Figure 1b** we only show the clade in south-west Galicia at the higher level (14 clusters).

Autonomous community	Proportion	Odds Ratio (95%CI) compared to overall	*p*-value (Fisher exact)
**Andalucía**	0.696	**0.412 (0.271 - 0.633)**	**$3.07x10^{-5}$**
**Aragón**	0.693	**0.434 (0.268 - 0.716)**	**$7.67x10^{-4}$**
Asturias	0.885	1.679 (0.498 - 8.844)	0.602
Baleares	0.750	0.643 (0.192 - 2.773)	0.505
**Cantabria**	0.556	**0.261 (0.091 - 0.774)**	**$7.38x10^{-3}$**
Castilla León	0.802	0.86 (0.502 - 1.534)	0.581
Castilla La Mancha	0.823	1.003 (0.501 - 2.187)	1.00
**Cataluña**	0.629	**0.226 (0.154 - 0.33)**	**$3.80x10^{-15}$**
**Valencia**	0.480	**0.158 (0.094 - 0.267)**	**$1.13x10^{-12}$**
Extremadura	0.806	0.898 (0.351 - 2.723)	0.811
Galicia	0.774	0.713 (0.416 - 1.265)	0.199
**Madrid**	0.333	**0.101 (0.031 - 0.297)**	**$4.77x10^{-6}$**
**Murcia**	0.491	**0.179 (0.097 - 0.33)**	**$1.18x10^{-8}$**
Navarra	0.839	1.129 (0.417 - 3.826)	1.00
País Vasco	0.829	1.053 (0.555 - 2.142)	1.00
La Rioja	0.900	1.969 (0.464 - 17.675)	0.555
Overall	0.822	.	.

Supplementary Table 6 | Proportion of individuals with distant grandparental birthplaces. For each autonomous community we consider all the individuals with at least one grandparent born in that region, and report the proportion of these individuals that we included in the maps because all four of their grandparents were born within 80Km of each other. We only include individuals with birthplace information for all four grandparents in this analysis. In each comparison with the overall proportion, the 'overall group' is made up of all the individuals not assigned to the autonomous community being tested. We highlight in bold the regions with fractions that differ significantly from the overall at the 0.01 significance level.

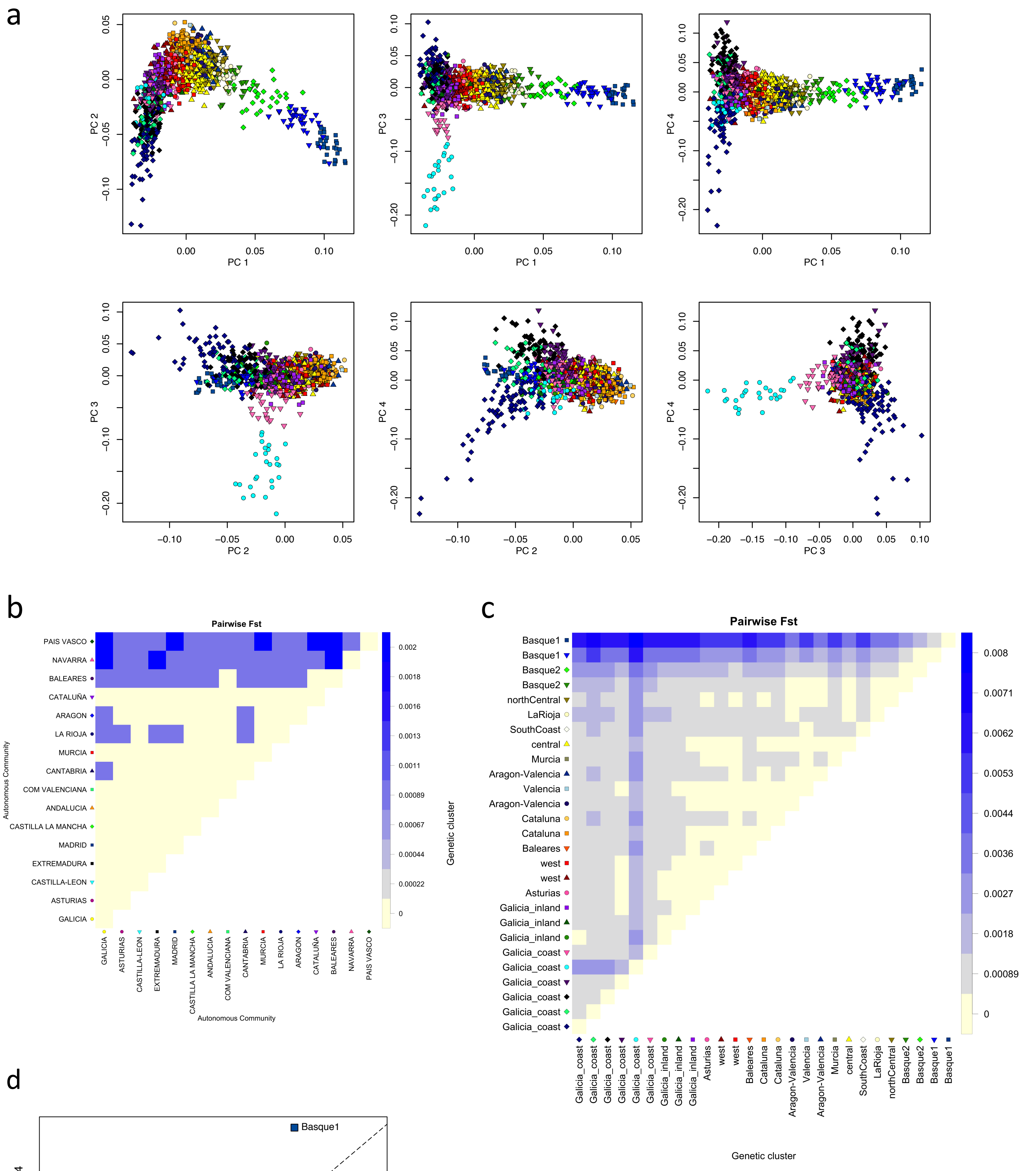

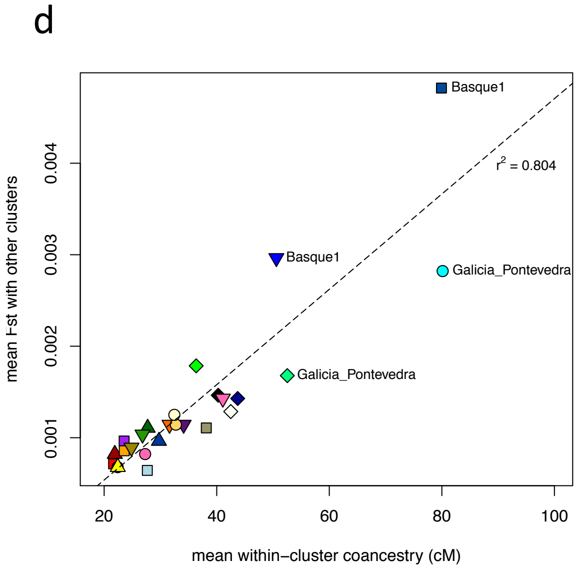

Supplementary Figure 9 | Relationship between fineSTRUCTURE clusters, F_{ST} and PCA. We computed F_{ST} and principal components using the genotype data at a set of LD-pruned SNPs (Methods). fineSTRUCTURE clusters are the same as those shown as points in **Figure 1.** **(a)** PCs 1-4 for of all Spanish individuals with points coloured according to fineSTRUCTURE clusters (see (c) or **Figure 1** for labels). **(b) and (c)** Pairwise F_{ST} when grouping samples by autonomous community (left) and fineSTRUCTURE clusters (right). **(d)** Correlation between F_{ST} and within-cluster coancestry as measured by fineSTRUCTURE. The *x*-axis shows the average within-cluster coancestry value for each cluster from 200 bootstrap re-samples of equal-sized clusters (Methods).

Supplementary References

1. Korn, J.M., et al., *Integrated genotype calling and association analysis of SNPs, common copy number polymorphisms and rare CNVs.* Nat Genet, 2008. **40**(10): p. 1253-60.
2. Manichaikul, A., et al., *Robust relationship inference in genome-wide association studies.* Bioinformatics, 2010. **26**(22): p. 2867-73.
3. Delaneau, O., J.F. Zagury, and J. Marchini, *Improved whole-chromosome phasing for disease and population genetic studies.* Nat Methods, 2013. **10**(1): p. 5-6.
4. The 1000 Genomes Project Consortium, *A map of human genome variation from population-scale sequencing.* Nature, 2010. **467**(7319): p. 1061--1073.
5. Purcell, S., et al., *PLINK: a toolset for whole genome association and population-based linkage analyses.* American Journal of Human Genetics, 2007. **81**.
6. Lawson, D.J., et al., *Inference of population structure using dense haplotype data.* PLoS Genet, 2012. **8**(1): p. e1002453.
7. Leslie, S., et al., *The fine-scale genetic structure of the British population.* Nature, 2015. **519**(7543): p. 309--314.
8. Fernandez-Rozadilla, C., et al., *A colorectal cancer genome-wide association study in a Spanish cohort identifies two variants associated with colorectal cancer risk at 1p33 and 8p12.* BMC Genomics, 2013. **14**: p. 55.
9. Pebesma, E.J. and R.S. Bivand, *Classes and methods for spatial data in R.* R News, 2005. **5**(2): p. 9--13.
10. Mackenzie, D., *Encyclopedia of the Languages of Europe*, G. Price, Editor. 2000, Blackwell Publishing.
11. Sánchez Pardo, J.C., *Territorio y poblamiento en Galicia entre la antigüedad y la plena Edad Media : tesis doctoral*. 2008, Universidade de Santiago de Compostela.
12. Instituto Nacional de Estadística *Population and Housing Censuses 2011*.
13. Varela, T.A., R.L. Aínsua, and J. Fariña, *Evolution of consanguinity in the Bishopric of Lugo (Spain) from 1900 to 1979.* Ann Hum Biol, 2001. **28**(5): p. 575--588.
14. Varela, T.A., R.L. Aínsua, and J. Fariña, *Consanguinity in the Bishopric of Ourense (Galicia, Spain) from 1900 to 1979.* Annals of Human Biology, 2003. **30**(4): p. 419-433.
15. Brion, M., et al., *Micro-geographical differentiation in Northern Iberia revealed by Y-chromosomal DNA analysis.* Gene, 2004. **329**: p. 17-25.
16. Varela, T.A., et al., *Gene flow and genetic structure in the Galician population (NW Spain) according to Alu insertions.* BMC Genet, 2008. **9**: p. 79.
17. Calderon, R., et al., *GM and KM immunoglobulin allotypes in the Galician population: new insights into the peopling of the Iberian Peninsula.* BMC Genet, 2007. **8**: p. 37.
18. Antonio Salas1, D.C., Maŕ ıa Victoria Lareu Jaume Bertranpetit and A. Carracedo, *mtDNA analysis of the Galician population: a genetic edge of European variation.* European Journal of Human Genetics, 1998.

19. R Core Team, *R: A Language and Environment for Statistical Computing*. 2014, R Foundation for Statistical Computing: Vienna, Austria.
20. Hellenthal, G., et al., *A genetic atlas of human admixture history.* Science, 2014. **343**(6172): p. 747-51.
